# Long-Term Use of Oral Bisphosphonates and Fracture Risk in Men with Traumatic Spinal Cord Injury

**DOI:** 10.33696/rehabilitation.2.013

**Published:** 2020

**Authors:** Laura D. Carbone, Beverly Gonzalez, Scott Miskevics, Cara Ray, Bella Etingen, Marylou Guihan, B. Catharine Craven, Frances M. Weaver

**Affiliations:** 1 Charlie Norwood Veterans Affairs Medical Center, Augusta, GA, USA; 2 Department of Medicine, Division of Rheumatology, J. Harold Harrison, MD, Distinguished University Chair in Rheumatology, Medical College of Georgia at Augusta University, Augusta, GA, USA; 3 Center of Innovation for Complex Chronic Healthcare, Edward J. Hines, Jr. VA Hospital, Hines, IL USA; 4 Feinberg School of Medicine, Northwestern University, Chicago, IL USA; 5 Department of Biostatistics, University of Illinois, Chicago, IL USA; 6 Department of Mathematics, Northeastern Illinois University, Chicago, IL USA; 7 Department of Medicine, University Health Network, University of Toronto, Toronto, ON, Canada; 8 Public Health Sciences, Stritch School of Medicine, Loyola University, Maywood, IL, USA

Lower extremity fractures in individuals with a spinal cord injury (SCI) cause significant morbidity [1] and contribute to excess mortality [2]. Early identification of persons at highest risk for fracture is possible using bone mineral density (BMD) testing by dual-energy X-ray absorptiometry (DXA) imaging [3]. Recent guidelines by the International Society for Clinical Densitometry (ISCD) recommend that all adults with a SCI resulting in permanent motor or sensory dysfunction should have a DXA scan of the total hip, proximal tibia, and distal femur as soon as they are medically stable [4], in order to assess bone health and fracture risk. A number of demographic and SCI-related factors, comorbidities and use of certain medications can also be used to determine those at highest risk for fracture [5]. A recent study reported that providers who care for patients with a SCI do utilize these BMD-related and clinical risk factors to identify subgroups of patients at highest risk for these events and to target pharmacological therapies for fracture prevention accordingly [6].

There are substantial differences in the pathophysiology of SCI-related bone loss compared with senile or postmenopausal osteoporosis [7–9]. It is not clear whether pharmacological therapies that are efficacious to treat senile osteoporosis and prevent fractures in the able-bodied population [10–12] are also effective in sublesional osteoporosis. In a cohort of Veterans with a chronic SCI, we recently reported that bisphosphonates, which may reduce fracture risk by upwards of 40% in the elderly able-bodied population [13] were not significantly associated with decreased lower extremity fracture risk [14]. However, in that report [14], we did not examine the relationship of long-term use of bisphosphonates with incident fractures. Sufficient duration of bisphosphonate use may be an important consideration for fracture risk reduction. In fact, among able-bodied postmenopausal women, transiliac bone biopsy data suggest that reductions in cortical porosity, a major determinant of bone strength [15,16], occur only after using alendronate for two years or more [17]. Moreover, studies that examine the efficacy of medications to prevent fracture are generally designed to include at least 36 months of therapy [11,12]. The goal of the current analysis was to examine the association of duration of use of oral bisphosphonates with risk of lower extremity (LE) fractures in male Veterans with a SCI.

## Methods

Men with a traumatic SCI were identified from the 2016 Veterans Health Administration (VHA) Allocation Resource Center (ARC) list using ICD-9 codes for SCI and treatment in a VHA SCI bed section or outpatient clinic [18]. ARC is a cumulative list of Veterans who have ever received healthcare from a VHA facility. Creation of a cohort of Veterans with traumatic SCI was described in an earlier publication [14]. VHA pharmacy data files were used to identify filled prescriptions for a Food and Drug Administration (FDA) approved oral bisphosphonates for osteoporosis (alendronate, ibandronate, or risedronate).

A matched nested case control study was performed. Cases were defined as men who were adherent to oral bisphosphonate therapy (defined as at least 80% of prescriptions refilled, calculated as medication possession ratio (MPR)) over a period of two years or more [19]. The date of a case’s first prescription served as his index date. Controls were defined as men who did not receive any FDA approved drug therapy for osteoporosis during the study period. The date of the control’s closest outpatient encounter to the case’s index date (within 365 days), served as his index date.

### Covariates

A number of important risk factors including demographics (age at index date, race [white, black, other, or missing]), medication use (prescriptions within 90 days of the index date for anticonvulsants, opioids or corticosteroids), prevalent LE fractures (FY2005-FY2010) [21] and SCI-related characteristics: duration of injury (acute [≤ 2 years], chronic [>2 years] or missing) level of injury (paraplegia, tetraplegia or missing), and completeness of injury (complete, incomplete or missing) [5] and having had a prior DXA [20], were considered in the analyses.

## Statistical Analyses

Cases and controls were censored at last date of follow-up within the study period, or fracture (first incident LE fracture (ICD-9 codes 808, 820–829) or pathological LE fracture (ICD-9 codes 733.10, 733.14–733.16, 733.19), or death. To reduce the effect of medical coding variability, we considered LE fracture ICD-9 codes with the same first three digits occurring within 120 days of each other to be the same fracture, consistent with previous research [21]. Cases were also censored if they were switched to another FDA approved osteoporosis medication. Within each nested cohort, each case was matched up to four controls using incidence density sampling. This allowed for cases and controls to be matched only on person time at risk of fracturing to minimize bias, contrary to our previous study where we matched on both person time at risk and propensity quintiles because we had a larger sample [14].

Conditional logistic regressions were used to examine relevant outcomes, which permitted unbiased estimates and 95% confidence intervals of odds ratios of incident LE fracture. Results were also confirmed via stratified Cox regression analyses and we report Hazard Ratios and 95% confidence intervals of the Hazard Ratios. Regressions were adjusted for covariates demonstrated to be statistically significant different at baseline (see Table 1). These included age at index date, corticosteroid use within 90 days of index date, opioid use within 90 days of index date, extent of SCI, prior fracture, and whether there was a prior DXA done. All statistical analyses were performed using SAS 9.2 and STATA version 14 statistical packages. A p-value of <0.05 was considered statistically significant.

There were some missing data with respect to level (6.98%), extent (13.06%) and duration of SCI (5.97%) in men. To account for this, without losing information, we included another category labeled “missing.”

This study was reviewed and approved by the Institutional Review Boards at the two VA facilities where this research was conducted. A HIPAA waiver and a waiver of informed consent were obtained.

## Results

**Figure 1:**
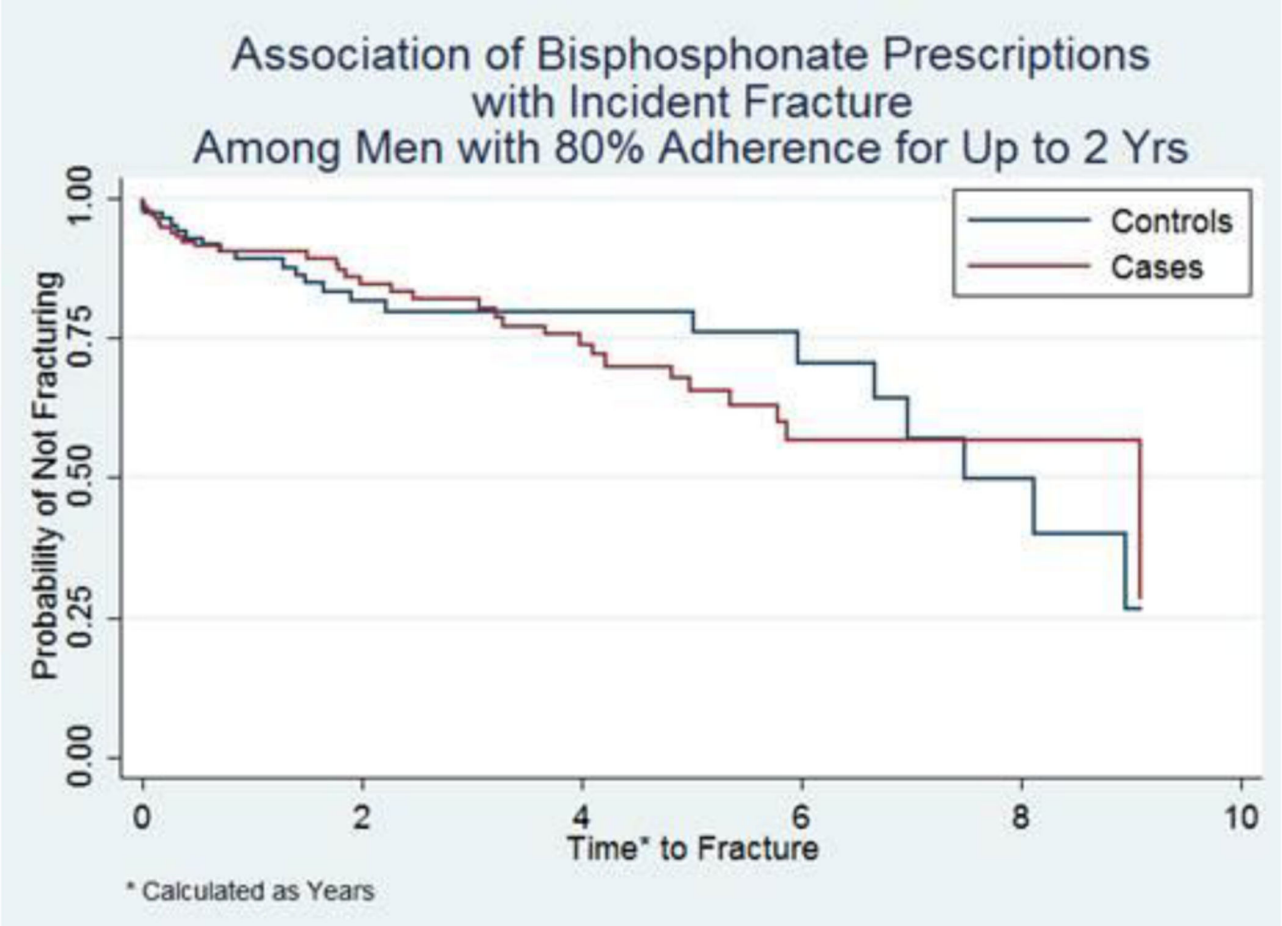
Life-table analysis to incident fracture in cases with oral bisphosphonate prescriptions who were at least 80% adherent to medication up to 2 years vs. controls.

We identified 7,989 male Veterans with a traumatic SCI who had utilized VHA health care between fiscal years (FY) 2010–2015 after applying the exclusions noted above. Of these, 267 had at least one filled prescription for an oral bisphosphonate. Among these, only 157 (58.80%) were adherent with this therapy for at least one year. Among these adherent cases, 65 were adherent with oral bisphosphonate therapy for at least two years (24 months), 42 were adherent for 24–36 months and 28 were adherent for 36 to 48 months. Baseline characteristics of the study population (at two years [24 months]) are shown in Table 1. There was no significant association of long-term use of oral bisphosphonates (defined as use of up to 24 months) and incident LE fracture (HR 0.97 [95% CI 0.25 to 3.75]) (Figure 1). Similarly, there was no significant association of oral bisphosphonate use with incident LE fractures among the 42 individuals who were adherent for 3 years (HR 1.17 [95% CI 0.26 to 5.35]) or among the 28 who were adherent for four or more years (HR 1.02 [95% CI 0.13 to 7.89]).

## Discussion

We did not find a significant association between oral bisphosphonate therapy with incident LE fractures among men with a traumatic SCI, despite adherence with this therapy for at least two years, and in some cases, up to four years. However, few (n=267) in this cohort who had at least one filled prescription for an oral bisphosphonate who were adherent at one year (n=157) continued to be adherent at two years (n=65, 41.4%) and beyond 2 years (n=42, 26.8% at three, and n= 28, 17.8% at four years).

Prevention of the first sentinel fracture event is important in persons with a SCI, not only because of its potential complications [1,2], but also because these fractures are associated with upwards of a fourfold increased risk of additional fractures over time [5]. Reports of the relationship of oral bisphosphonate use with positive changes in BMD, a central predictor of fractures in persons with a SCI [3], are inconsistent [22]. This report extends our prior work, which revealed no overall association of filled prescriptions for bisphosphonates with incident fractures [14], to suggest that, even with long-term adherence to oral bisphosphonate therapies, use of these medications is not associated with risk of LE fractures in men with a traumatic SCI.

We found that there was poor adherence over time with oral bisphosphonate therapy in our cohort of male Veterans with a traumatic SCI. This finding is aligned with prior reports of low adherence to oral bisphosphonate therapies for fracture prevention in the able-bodied population [23]. We have previously reported in a case series, that side effects, predominantly gastrointestinal, were the main reason for discontinuation of oral bisphosphonates in Veterans with a SCI [24].

There are a number of limitations to this work. First, the number of individuals in our cohort who were adherent with oral bisphosphonate use over time was limited. Additionally, administrative databases do not contain information on whether the medications were actually taken and if taken, were used correctly, which is an important consideration, as oral bisphosphonates are very poorly absorbed if taken with food or beverages other than water [25].

In conclusion, in men with a traumatic SCI, long-term adherence with bisphosphonate prescription refills is not associated with reductions in lower extremity fractures. New therapies to prevent fractures in individuals with a SCI are needed.

## Figures and Tables

**Table 1:** Baseline characteristics of study population by filled prescriptions for oral bisphosphonates and 24 month or greater adherence.

DEMOGRAPHIC AND CLINICAL CHARACTERISTICS	CASES Oral Bisphosphonate Script+ (n=65)	CONTROLS No Osteoporosis Scripts+ + (n=7,722)	P-Value
*Age (mean, sd) ≤ 50 > 50	57.09 ± 13.79 12 (18.46) 53 (81.54)	55.14 ± 14.06 2,528 (32.74%) 5,194 (67.26)	0.02
**Race (%)** White Black Other	45 (69.23%) 10 (15.38%) 10 (15.38%)	5,124 (66.36%) 1,618 (20.95%) 980 (12.69%)	0.50
**Anticonvulsant use (%)**	3 (4.62%)	257 (3.33%)	0.48
**Corticosteroid use (%)**	4 (6.15%)	94 (1.22%)	0.009
**Opioid use (%)**	7 (10.77%)	300 (3.89%)	0.01
**SCI CHARACTERISTICS**			
**Level of injury (%)** Paraplegia Tetraplegia Missing	29 (44.62%) 31 (47.69%) 5 (7.69%)	3,434 (44.47%) 3,749 (48.55%) 539 (6.98%)	0.97
**Extent of injury (%)** Complete Incomplete Missing	34 (52.31%) 21 (32.31%) 10 (15.38%)	2,965 (38.40%) 3,750 (48.56%) 1,007 (13.04%)	0.03
**Prevalent Lower Extremity Fractures (%)**	11 (16.92%)	4 (0.05)	<0.0001
**Duration of SCI Related Injury (%)** Acute Chronic Missing	12 (18.46 %) 49 (75.38%) 4 (6.15%)	1,375 (17.81 %) 5,886 (76.22%) 461 (5.97%)	0.99
**DXA prior to Index Date (%)**	45 (69.23%)	875 (11.33%)	<0.0001

+ Patient with prescription for alendronate, ibandronate, or risedronate

* Age at time of prescription or index date

P-Value from Chi-Square or from Fisher’s Exact Test
